# Bisphosphonates regulate cell growth and gene expression in the UMR 106-01 clonal rat osteosarcoma cell line

**DOI:** 10.1054/bjoc.2000.1679

**Published:** 2001-04

**Authors:** P S Mackie, J L Fisher, H Zhou, P F M Choong

**Affiliations:** Department of Orthopedics and; Department of Medicine, St. Vincent's Hospital, Fitzroy, Melbourne, Victoria, 3065, Australia

**Keywords:** osteosarcoma, bisphosphonates, apoptosis, osteopontin, RANKL, osteoprotegerin

## Abstract

Local growth of osteosarcoma involves destruction of host bone by proteolytic mechanisms and/or host osteoclast activation. Osteoclast formation and activity are regulated by osteoblast-derived factors such as the osteoclast differentiating factor, receptor activator of NF-κB ligand (RANKL) and the inhibitor osteoprotegerin (OPG). We have investigated the in vitro effects of bisphosphonates on a clonal rat osteosarcoma cell line. The aminobisphosphonate pamidronate was added to UMR 106-01 cell cultures (10^−8^M to 10^−4^M up to 5 days). The non-aminobisphosphonate clodronate was administered for the same time periods (10^−6^M to 10^−2^M). Cell proliferation, apoptosis and mRNA expression was assessed. Both agents inhibited cell proliferation in a time- and dose-dependent manner. ELISA analysis demonstrated an increase in DNA fragmentation although there was no significant dose-related difference between the doses studied. Bisphosphonate-treated cultures had a greater subpopulation of cells exhibiting morphological changes of apoptosis. Expression of mRNA for osteopontin and RANKL was down-regulated by both agents, while the expression of mRNA for alkaline phosphatase, pro-α1(I) collagen and OPG was not altered. Out in vitro work suggests the bisphosphonates not only have direct effects on osteosarcoma cell growth and apoptosis, but also, by altering the relative expression of osteoclast-regulating factors, they may inhibit the activity of osteoclasts and their recruitment. © 2001 Cancer Research Campaignhttp://www.bjcancer.com


*British Journal of Cancer* (2001) **84**(7), 951–958
© 2001 Cancer Research Campaign
doi: 10.1054/ bjoc.2001.1679, available online at http://www.idealibrary.com on IDEAL®

http://www.bjcancer.com

### Bisphosphonates regulate cell growth and gene expression in the UMR 106-01 clonal rat osteosarcoma cell line

**PS Mackie[1], JL Fisher[1], H Zhou[2] and PFM Choong[1]**

[1]Department of Orthopedics and [2]Department of Medicine, St. Vincent's Hospital, Fitzroy, Melbourne, Victoria 3065, Australia

**Summary** Local growth of osteosarcoma involves destruction of host bone by proteolytic mechanisms and/or host osteoclast activation. Osteoclast formation and activity are regulated by osteoblast-derived factors such as the osteoclast differentiating factor, receptor activator of NF-κB ligand (RANKL) and the inhibitor osteoprotegerin (OPG). We have investigated the in vitro effects of bisphosphonates on a clonal rat osteosarcoma cell line. The aminobisphosphonate pamidronate was added to UMR 106-01 cell cultures ($10^{-8}$ M to $10^{-4}$ M up to 5 days). The non-aminobisphosphonate clodronate was administered for the same time periods ($10^{-6}$M to $10^{-2}$ M). Cell proliferation, apoptosis and mRNA expression was assessed. Both agents inhibited cell proliferation in a time- and dose-dependent manner. ELISA analysis demonstrated an increase in DNA fragmentation although there was no significant dose-related difference between the doses studied. Bisphosphonate-treated cultures had a greater subpopulation of cells exhibiting morphological changes of apoptosis. Expression of mRNA for osteopontin and RANKL was down-regulated by both agents, while the expression of mRNA for alkaline phosphatase, pro-α1(I) collagen and OPG was not altered. Out in vitro work suggests the bisphosphonates not only have direct effects on osteosarcoma cell growth and apoptosis, but also, by altering the relative expression of osteoclast-regulating factors, they may inhibit the activity of osteoclasts and their recruitment. © 2001 Cancer Research Campaign http://www.bjcancer.com

**Keywords**: osteosarcoma; bisphosphonates; apoptosis; osteopontin; RANKL; osteoprotegerin

Despite significant improvements in patient survival and local disease control, 25% of patients with osteosarcoma develop metastases and the surgery for many extremity lesions still includes amputation. Thus, modalities that inhibit tumour growth and the metastatic cascade will have a significant impact on patient survival and potential for limb sparing surgery.

Tumours induce bone destruction by cellular processes, such as osteoclast-mediated bone lysis. The importance of osteoclast-mediated lysis has been demonstrated in other malignancies, where the development of osseous metastases has been shown to be mediated by soluble tumour-related osteoclast activating factors (Galasko, 1976). More recently, the induction of specific osteoclast recruiting factors in osteoblasts/stromal cells following contact with myeloma and breast cancer cells has been demonstrated (Chikatsu et al, 2000). Bisphosphonates have recognized efficacy in reducing bone destruction, pain and pathological fracture in a variety of lytic primary and metastatic diseases of the skeleton (Thiébaud et al, 1991; Coleman and Purohit, 1993; Shipman et al, 1997; Bloomfield, 1998; Diel et al, 1998). They have more recently been shown to inhibit establishment and growth of prostate cancer metastases, which are generally considered to be osteoblastic in their growth pattern (Adami, 1997). We proposed that, as with the intraosseous growth of prostate cancer, the local spread of osteosarcoma involves proteolytic and osteoclast-mediated bone destruction. The bisphosphonates may reduce bone destruction by uncoupling close regulation of osteoclast activity by osteoblast-like cells.

The aims of our study were to examine the effects on the regulation of proliferation and apoptosis by bisphosphonate treatment of a clonal osteosarcoma cell line. Further, we assessed the expression of an osteoclast differentiating factor, receptor activator of NF-κβ (RANKL) (Yasuda et al, 1998b) and an osteoclastogenesis inhibitory factor, osteoprotegerin (OPG) (Simonet et al, 1997; Yasuda et al, 1998a). Finally, we investigated the effects on osteoblast-related gene expression by an aminobisphosphonate and a non-aminobisphosphonate. The use of a bisphosphonate from each group allows comparison of their relative potencies with the known effects on osteoclasts and bone resorption studies (Fleisch, 1993).

### MATERIALS AND METHODS

### Cell culture

UMR 106-01 cells derived from a $^{32}$P-induced osteosarcoma in rats (Martin et al, 1976) were used. Cells were cultured in 75 $cm^2$ tissue culture flasks (Greiner Cellstar) in α-modified Minimal Essential Medium (α-MEM) containing hepes 4 g $l^{-1}$, sodium bicarbonate 1.95 g $6^{-1}$, gentamicin 80 mg $6^{-1}$, pH 7.4 and 10% fetal bovine serum (FBS), incubated at 37°C and equilibrated in 5% $CO_2$ in air. Subcultures were performed using 0.0125% trypsin in 0.5 mM $Na_2$EDTA in calcium and magnesium-free phosphate buffer (1 × versene) to harvest the cells.

*Received 17 August 2000*
*Revised 3 January 2001*
*Accepted 4 January 2001*

*Correspondence to:* PFM Choong

### Bisphosphonates

The aminobisphosphonate pamidronate (Aredia, Novartis Pharmaceuticals, Australia) and non-aminobisphosphonate clodronate (provided by Prof TJ Martin of St Vincent's Institute of Medical Research) were dissolved in sterile water and stored frozen in aliquots of $10^{-6}$ M to $10^{-2}$ M until use.

### Proliferation studies

Cell count / proliferation studies were performed on UMR 106-01 cells grown in monolayer in 6-well culture plates (10.06 $cm^2$ Nunc Products), with a plating density of 1000–1500 cells per well in α-MEM medium containing 10% FBS. Trypan blue was used to confirm that only cell populations with greater than 90% cell viability were used for seeding. Medium was replaced after 1 day with α-MEM containing 2% FBS for a further 24 hours prior to treatment of bisphosphonates, at concentrations of pamidronate $10^{-8}$ to $10^{-4}$M and clodronate $10^{-6}$ to $10^{-2}$M. Medium was replaced every 48 hours and bisphosphonates replenished with the new media, to ensure continual exposure of cells to a relatively uniform concentration of the bisphosphonates. Cells were harvested at various time points with 0.0125% trypsin in 1 × versene and were counted on a coulter counter (Model DN, Coulter Electronics, Bedfordshire, England). Experiments were repeated at least 3 times with 6 wells per treatment group within each experiment. Three separate coulter counts were performed on each well sample and the results compared against controls using Student's *t*-test.

### Apoptosis assessment

Apoptotic cell ratios in cell cultures were determined by ELISA quantification of DNA fragmentation (Boehringer Mannheim) according to the manufacturer's specifications. Within microtitre wells, $1 \times 10^4$ cells were incubated for 4 hours with serial concentrations of the two bisphosphonates. After centrifugation (to ensure detached apoptotic cells were retained) the cells were lysed and an aliquot of the supernatant transferred to streptavidin-coated ELISA microtitre wells and exposed to anti-histone biotin and anti-DNA POD solutions for 2 hours. Unbound antibodies were removed by washing and the fragmented DNA-POD immunocomplexes were detected photometrically, using ABTS (2,2′-Azino-di[3-ethylbenzthiazolin-sulphonate]) substrate. A microtitre plate reader (Titertek, Multiskan® Plus) was used to record the resultant color change (at 405 nm against substrate solution as a blank, wavelength 490 nm) and differences assessed using 1-way ANOVA and Fisher's PLSD analyses.

To confirm the ELISA findings, cultures were assessed for the characteristic morphological features of apoptosis. Cell cultures were grown in monolayer on 8 well chamber slides (Lab-Tek, Nunc Products) and exposed after 24 hours to the two bisphosphonates for 24 and 48 hours. As with the ELISA studies, slides were briefly centrifuged on microtitre plate carriers to return detached cells to the monolayer and the slides were fixed in 3% (v/v) acetic acid in 95% methanol for 1 minute before staining with haematoxylin and eosin. Three representative high-powered fields (400 ×) were analysed for the percentage of cells exhibiting apoptotic characteristics of nuclear pyknosis, apoptotic bodies, membrane blebbing and cytoplasmic inclusions. Results were recorded as an apoptotic subpopulation of less than 5%, 5 to 20%, or greater than 20%.

### Genotype studies

UMR 106-01 cells were seeded at $1 \times 10^4$ cells per 56 $cm^2$ plate (Falcon, Becton Dickinson) in α-MEM containing 10% FBS. Medium was replaced after 24 hours with α-MEM containing 2% FBS to reduce effects of endogenous cytokines on cell growth. Bisphosphonate treatment was not started until 24 hours after this change and media and bisphosphonates ware replenished every 48 hours. Bisphosphonate was added at concentrations of between $1 \times 10^{-8}$ and $1 \times 10^{-4}$ M for pamidronate and $1 \times 10^{-6}$ and $1 \times 10^{-2}$ M for clodronate over time frames of between 6 days and 4 hours, prior to confluence of control cultures. Previous experiments on the UMR 106-01 cells line allowed characterization of the culture growth pattern and accurate prediction of times to control cell culture confluence. All times and concentrations were assessed in triplicate.

Total RNA was isolated from the UMR 106-01 cultures using TRIZOL (Gibco PRL, Life Technologies) according to the manufacturer's instructions and redissolved in RNase-free TE Buffer, pH 8.0, to a concentration of 2 μg $\mu l^{-1}$ and stored at – 80˚C. As total RNA from each culture was diluted to a standard 2 μg $\mu l^{-1}$, prior to agarose gel electophoresis, the resultant Northern hybridization studies allow comparative analysis of mRNA as a percentage of total RNA in each treatment group.

### Synthesis of riboprobes

Probes were labelled with digoxigenin (DIG) using an RNA labeling kit (Boehringer Mannheim GmbH, Mannheim, Germany) according to the manufacturer's instructions.

A 2.4 kb *Eco*RI fragment of rat alkaline phosphatase (ALP) cDNA, Dr. G. Rodan, Merck, Sharp and Dohme Research Laboratories, West Point, PA, was further cut with *Bam*H1 and the 1.1 kb *Eco*R1-*Bam*H1 fragment was subcloned into Stratagene pBluescript plasmid (pBS). The plasmid was linearized with *Bam*HI and transcribed with T7 RNA polymerase to generate a 1.1-kb antisense riboprobe. A 500 bp *Eco*RI fragment of rat bone GLA protein/osteocalcin (BGP) cDNA (Dr J Wozney, Genetics Institute, Cambridge, MA) was subcloned into pBS plasmid vector and then linearized with *Hind*III before being transcribed into the antisense strand with T3 RNA polymerase. Rat matrix GLA protein (MGP) cDNA (Dr P Price, University of San Diego, La Jolla, CA) was subcloned into pBS. The plasmid was linearized with *Hind*III and transcribed with T3 to produce an antisense riboprobe. A 1.6 kb *Xba*I-*Xho*I insert of human osteopontin (OPN) cDNA in pBS (Dr L Fisher, National Institute of Dental Research, USA) was linearized with *Xba*I and transcribed with T7 polymerase to generate the antisense riboprobe. Pro-α1(I) collagen RNA riboprobe was obtained by subcloning a 1.6 kb Pst I fragment of rat pro-α1(I) collagen cDNA (Dr J Bateman, Royal Children's Hospital, Melbourne, Australia) into the pSPT 19. This plasmid vector was linearized using *Eco*RI and was then transcribed with T7 RNA polymerase to generate a 1.6 kb antisense riboprobe. A 750 bp RANKL cDNA fragment, (bp 381–1130) (Wong et al, 1997) and a 545 bp OPG cDNA fragment (bp 778–1323) (Simonet et al, 1997) were obtained from N Horwood and R Thomas, St Vincent's Institute of Medical Research, Melbourne, Australia. Each reverse transcription polymerase chain reaction product was cloned into pGEM-T, linearized with *Nde*I and transcribed with T7 RNA polymerase to generate the respective antisense riboprobes (Kartsogiannis et al, 1999).

*© 2001 Cancer Research Campaign*

### Northern blot analysis

Total RNA was loaded at 20 μg per lane and separated overnight in a 1.5% agarose-formaldehyde gel. The RNA was transferred by vacuum suction to nylon hybridization filters (Genescreen, NEN Life Science Products) hybridized overnight in a buffer solution (formamide 50%, 5 × SSC, block reagent 2% (Boehringer Mannheim), SDS 0.02%) with DIG labeled riboprobes at a concentration of 25 ng $ml^{-1}$. Probes for OPN, ALP, Pro-α1 (I) collagen, BGP, MGP, RANKL and OPG were used. Sequential washes (2 × SSC / 0.1% SDS, 2 washes of 5 minutes at room temperature, 2 washes at 65˚C in 0.1 × SSC / 0.1% SDS). The hybridized probes were subsequently detected with anti-DIG antibody coupled to alkaline phosphatase (Boehringer Mannheim) and visualized by chemiluminescence autoradiographic detection with CDP-Star (Boehringer Mannheim).

Filters were stripped of probe by washing in DEPC-treated $H_2O$ followed by 15 minutes washing in 50% formamide, 50 nM Tris-HCl (pH8.0) and 1% SDS at 100˚C, and stored in 2 × SSC. Hybridized probes were normalized against a DIG-labelled 18 S oligonucleotide probe (amino acid (180–156) sequence: CGG CAT GTA TTA GCT CTA GAA TTA CCA CAG) (Chan et al, 1984) and detected with Anti-DIG as described above.

mRNA was quantitated by densitometry (ImageQuaNT software, Molecular Dynamics), normalizing each probe expression against 18 S expression on the same filter, and repeated with at least 3 filters for each probe.

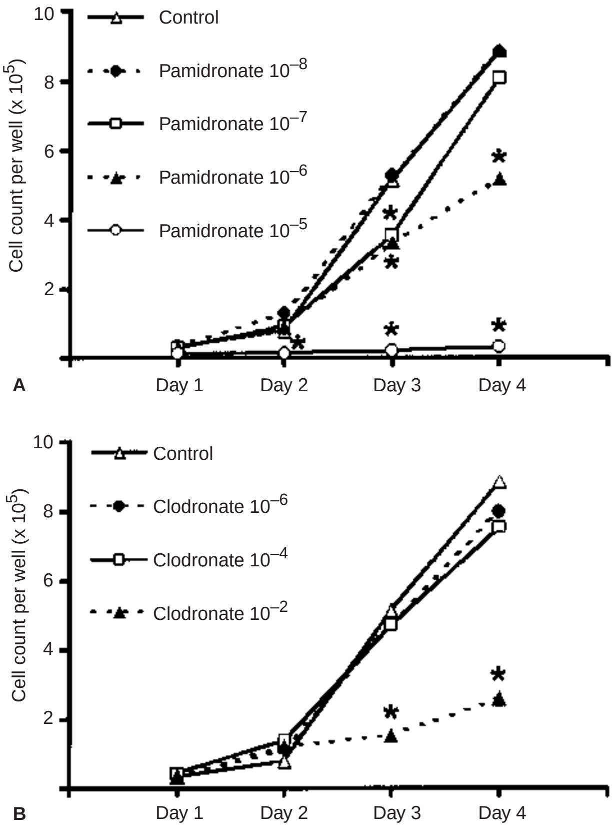

**Figure 1** The effect of bisphosphonate exposure on proliferation of UMR 106–01 cells grown in monolayer. Cells were detached by trypsinization and counted by coulter counter on the days indicated. Points are mean of three samples. Experiments were performed more than three times with similar results and low standard error of means (not discernible at this resolution). (**A**) – Pamidronate exposure. $10^{-8}$ M to $10^{-5}$ M. (**B**) – Clodronate exposure. $10^{-6}$ M to $10^{-2}$ M. (*) = *P*-value < 0.05 compared against control

### RESULTS

### Cell proliferation and cell death study

There was a dose-related inhibition of cellular proliferation of UMR 106–01 cells in monolayer culture with both bisphosphonates used. At $1 \times 10^{-4}$ M pamidronate exposure and > $1 \times 10^{-2}$ M clodronate exposure marked cytotoxicity was noted, with death of all cells within 24 to 48 hours (results not shown). Inhibition of growth occurred at concentrations of pamidronate between $1 \times 10^{-6}$ M and $1 \times 10^{-5}$ M and clodronate between $1 \times 10^{-2}$ M and $1 \times 10^{-2}$ M (Figures 1A, 1B).

DNA internucleosomal fragmentation, detected by ELISA utilizing anti-DNA and anti-histone antibodies, increased more than 3-fold in cell cultures exposed for 4 hours to $10^{-4}$ M pamidronate (Figures 2A, 2B). Fragmentation at pamidronate concentrations of $10^{-5}$ M to $10^{-9}$ M and with clodronate concentrations of $10^{-3}$ M and $10^{-5}$ M was greater than in control cultures ($P < 0.05$). Using 1-way ANOVA analysis no statistical dose-dependent difference was noted between the concentrations studied within each treatment group.

Examination of cells treated at $1 \times 10^{-5}$ to $1 \times 10^{-4}$ M of pamidronate by phase contrast microscopy demonstrated large numbers of non-viable cells within the cultures. Large numbers of cells were seen exhibiting intracytoplasmic inclusions, loss of cell shape, loss of cell to cell and cell to surface adhesion, and the presence of apoptotic bodies (Figures 3A, 3B). Visual quantification of the percentage of cells exhibiting these features demonstrated an increase in the proportion of apoptotic cells in cultures exposed to higher bisphosphonate concentrations (Table 1). Exposure to clodronate at doses approximately 100-fold higher than those of pamidronate produced similar morphological features of apoptosis within the osteosarcoma cultures. These features of cytotoxicity were not thought to be due to the calcium-chelating effect of bisphosphonates, as cultures exposed to equimolar concentrations of EDTA did not produce the same effect (results not shown).

### Gene expression

At $1 \times 10^{-5}$ M pamidronate the expression of OPG was unaltered over treatment periods of 0 to 5 days when normalized against 18 S expression on the same filter. RANKL mRNA expression was down regulated by prolonged exposure of the cells to pamidronate, when normalized against 18 S and hybridizing equal amounts of total sample RNA compared with control RNA (Figure 4A, 4B).

There was a significant reduction in OPN mRNA expression, again when normalized against 18 S on the same filters and compared with equal control total RNA hybridization. We noted no change in the expression of ALP or pro-α1 (I) collagen mRNA (Figures 4A, 4C). Similar effects on mRNA expression for the above proteins were observed in cells treated with clodronate, albeit at a concentration 100-fold that of pamidronate (results not shown). BGP and MGP are expressed in some osteoblast-like cell lines however there is no inherent expression of mRNA for these matrix proteins by UMR106-01 cells and exposure to bisphosphonate did not alter this.

© 2001 Cancer Research Campaign

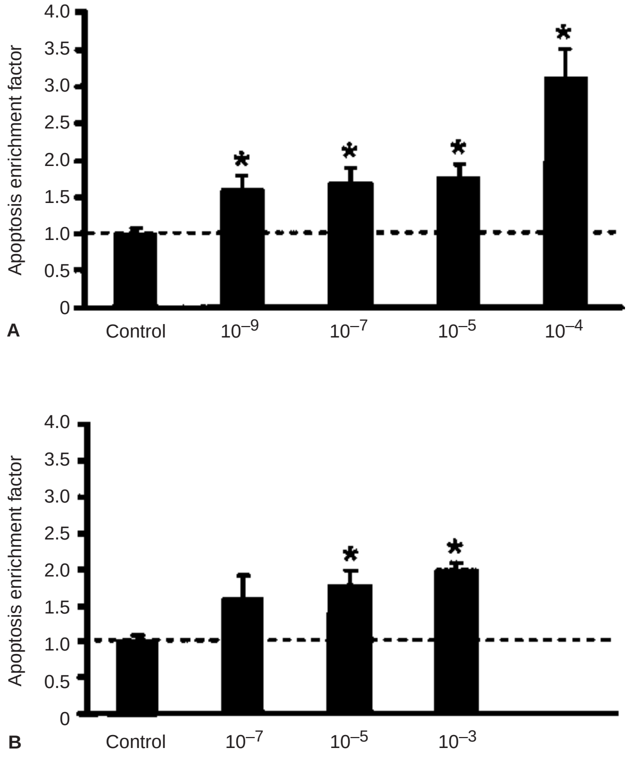

**Figure 2** Assessment of apoptosis after 4 hours of bisphosphonate exposure to $1 \times 10^4$ UMR 106–01 cells. The apoptotic subpopulation was detected by ELISA photometric assay (405 nm against substrate solution approx 490 nm) and the enrichment factor for apoptosis compared with untreated cells graphically shown. Error bars represent SE mean over three experiments. 2(**A**) – Pamidronate exposure $10^{-9}$, $10^{-7}$, $10^{-5}$, $10^{-4}$ M. 2(**B**) – Clodronate exposure $10^{-7}$, $10^{-5}$, $10^{-3}$ M. (*) = *P*-value < 0.05 compared against control.

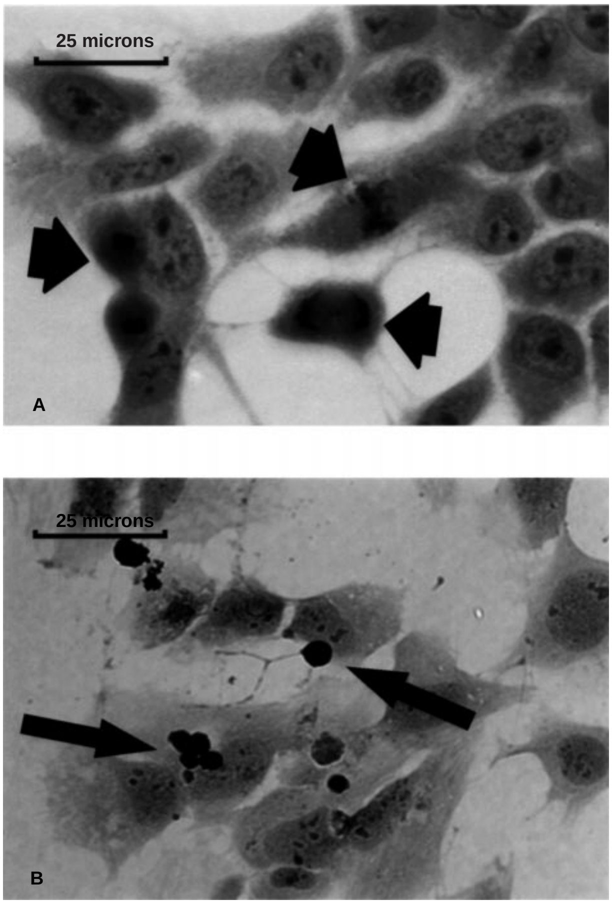

**Figure 3** Representative microphotographs of UMR 106–01 cells grown in monolayer, fixed with acetic acid 3% v.v. in methanol and stained with hematoxylin and cosin. (**A**) – Untreated cells with distinct cellular structure and frequent mitoses (arrow heads) × 400 (**B**) – Following 24 hours of pamidronate ($10^{-5}$ M) apoptotic cells are more frequent, with many apoptotic bodies developing from nuclear fragmentation (arrows) × 400

Exposure of the cultures to concentrations of pamidronate < $10^{-5}$ M and clodronate < $10^{-3}$ M did not significantly alter mRNA expression, as detected by Northern hybridization (results not shown).

### DISCUSSION

To study the effects of bisphosphonates on osteosarcoma cells in vitro we have utilized the UMR 106-01 cell line. This cell line is very well characterized and has been used, in our laboratory, to induce reproducible and representative tibial osteosarcomas in nude mice (unpublished). We have shown that pamidronate and clodronate have a dose- and time-dependent inhibitory effect on monolayer growth of osteosarcoma cell. Studies on the effect of bisphosphonates on osteoblasts have differed in their findings although several have demonstrated inhibitory effects at higher doses (Evans and Braidman, 1994; Goziotis et al, 1995; García Moreno et al, 1998). Plotkin et al showed that bisphosphonates prevent osteoblast apoptosis using several criteria, however noted

**Table 1** Morphological assessment of apoptotic cells in monolayer on 8 well tissue culture microscopy slides. Three representative high-power fields were examined and the number of cells exhibiting characteristic features of apoptosis determined as a percentage of the total population. (<5% of cells apoptotic. 5–20 % of cells apoptotic. >20% of cells apoptotic

| Control | | Pamidronate | | | Clodronate | | |
|---|---|---|---|---|---|---|---|
| | | $10^{-9}$ M | $10^{-7}$ M | $10^{-5}$ M | $10^{-7}$ M | $10^{-5}$ M | $10^{-3}$ M |
| 24 Hours | <5% | < 5% | < 5% | < 5% | < 5% | < 5% | 5–20% |
| 48 Hours | <5% | < 5% | < 5% | 5–20% | < 5% | 5–5% | < 20% |

© 2001 Cancer Research Campaign

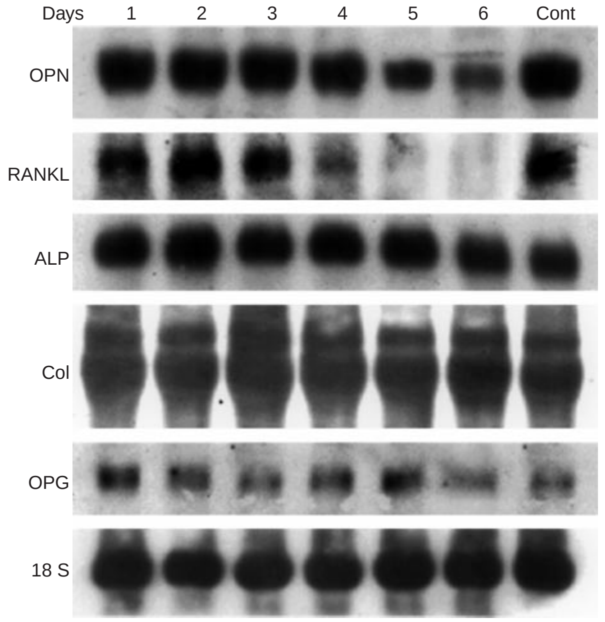

**Figure 4(A)** Detection of mRNA expression for bone-related proteins and osteoclast-regulating factors. UMR 106–01 osteosarcoma cell monolayer cultures were exposed to $10^{-5}$ M pamidronate for 1 to 6 days prior to confluence of controls. Total RNA (20 μg) was loaded in each lane, separated by gel electrophoresis and mRNA visualized as described in the text. 18S ribosomal detection was performed to normalize the amount of RNA present in each lane. Numbers above each lane represent number of days of pamidronate exposure. Control = untreated cells. OPN = osteopontin: RANKL = receptor activator of NF-κB ligand: ALP = alkaline phosphatase: Col = pro-α1(1) collagen: OPG = osteoprotegerin: 18 S = Housekeeping gene

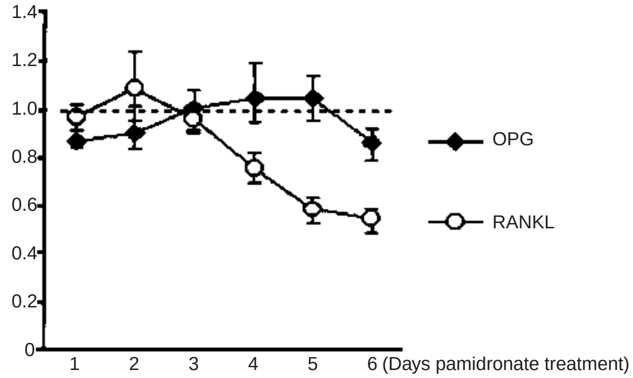

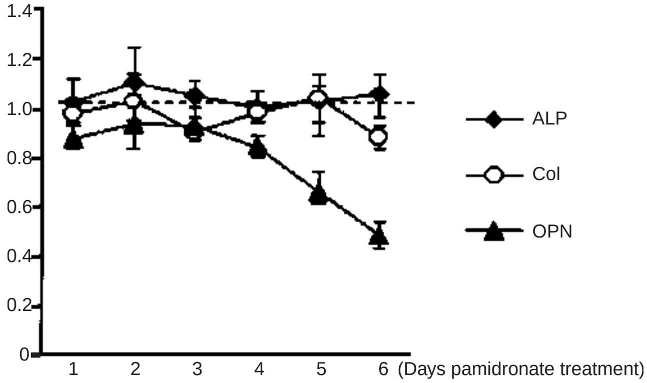

**Figure 4 (B and C)** Densitometric analysis of Northern hybridization for bone-related factors and osteoclast regulating genes. Filters with bound and visualized mRNA probes were analysed and normalized against the corresponding 18 S control bands for each filter. Y-axis represents the value expressed as a ratio of the normalized untreated control for the same time point and concentration. Average and range of three filters shown for each probe. (**B**) OPG = osteoprotegerin. RANKL = receptor activator of NF-κB ligand (**C**) ALP = alkaline phosphatase. Col = pro- α1(1) collagen. OPN = osteopontin a biphasic response, and were unable to resolve an increase in morphological signs of apoptosis seen in osteocytes exposed to bisphosphonates (Plotkin et al, 1999). An inhibitory effect was noted by Tan et al in $^{3}$H-thymidine uptake studies in UMR 106 osteosarcoma cells exposed to pamidronate (Tan et al, 1988), although the effect on cellular metabolic activity (rather than cell number) at $5 \times 10^{-4}$ M was assessed. Time-or concentration-dependent differences were not reported.

As observed in our study, pamidronate exposure of $>10^{-4}$ M was cytotoxic to the cell monolayer whereas at $1 \times 10^{-5}$ M pamidronate we noted an inhibition of proliferation and an increase in the proportion of cells in monolayer undergoing apoptosis. At this lower concentration we demonstrated no change over time in the expression of ALP or pro-α1 (I) collagen mRNA but there was progressive down regulation of OPN and RANKL mRNA expression. This selective alteration in mRNA expression suggests that pamidronate, at a dose of $1 \times 10^{-5}$ M, may regulate specific cell functions, such as proliferation and gene expression rather than inducing direct cytotoxicity. The dose at which effects on the osteosarcoma cell line were noted is higher than those used in many studies on osteoclasts, although similar concentrations have been demonstrated to cause apoptosis in breast cancer cell lines (pamidronate $>1 \times 10^{-5}$ M) (Senaratne et al, 2000) and myeloma cell line cultures (pamidronate $>1 \times 10^{-4}$ M) (Shipman et al, 1997). These findings may still be significant if translated to the in vivo setting, as the strong hydroxyapatite affinity of bisphosphonates is thought to drive their local concentration within the bone microenvironment significantly higher than the serum level. Novel studies by Sato et al demonstrated localization of radiolabelled alendronate beneath resorption lacuna at concentrations approaching $1 \times 10^{-3}$ M (Sato et al, 1991) and further in vitro assays demonstrated an inhibitory $IC_{50}$ of $10^{-6}$–$10^{-5}$ M for pamidronate in an ivory slice assay of osteoclast activity.

We have studied the effects of bisphosphonates on the expression of two factors by an osteosarcoma cell line which are known to be physiological regulators of osteoclast activity. The osteoclast differentiating factor (RANKL) (Yasuda et al, 1998b) and M-CSF (Yoshida et al, 1990) are required for osteoclast differentiation. Regulation of these two factors is thought to be the common final pathway by which the resorption-inducing agents such as parathyroid hormone, 1,25 dihydroxyvitamin $D_3$ and interleukins promote osteoclastogenesis (Horwood et al, 1998). The soluble TNF family member (OPG) acts as a decoy receptor for RANKL, and expression by osteoblasts under hormone regulation is thought to inhibit

*© 2001 Cancer Research Campaign*

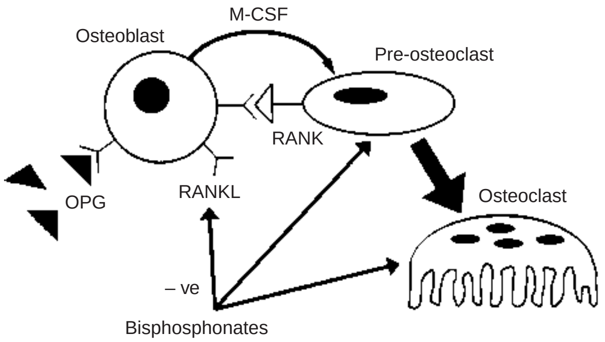

**Figure 5** Mechanisms via which the bisphosphonates may affect osteoclast function. Downregulation of RANKL mRNA expression in the presence of continued OPG mRNA production accounts for a net reduction in osteoclast differentiating signals from osteoblasts as the relatively abundant OPG saturates RANKL. RANKL binding to the receptor on osteoclast progenitor cells is thus inhibited. Direct apoptotic effects on mature osteoclasts are known and differentiation of osteoclasts is also thought to be directly suppressed.

osteoclastogenesis by binding the differentiating factor (Simonet et al, 1997; Yasuda et al, 1998a).

We found that OPG mRNA expression was unaltered by the bisphosphonates and RANKL expression was reduced. This down-regulation of RANKL mRNA expression suggests that interference with osteoclastogenesis by the bisphosphonates could be, in part, attributable to inhibition of the release of osteoclast differentiating factors by stromal cells or osteoblasts. It has recently been shown that the induction of osteoclastogenesis is dependent upon the relative abundance of RANKL compared with the levels of OPG (Horwood et al, 1998). The anticipated in vivo net effect of RANKL down-regulation but with stable OPG levels would be a reduction in local osteoclast recruitment or activation (Figure 5). The expression of mRNA for RANKL by osteosarcoma cells in vitro raises the possibility that the tumours may directly recruit osteoclasts at the tumour-bone interface in vivo, rather than acting on host stromal cells to induce osteoclast recruitment (as seen in metastatic skeletal malignancies). Alteration in the ratio of osteoclast recruiting and inhibiting factors following bisphosphonate exposure suggests these agents may have an effect on this paracrine recruitment.

Bisphosphonates exert their anti-resorptive effects via several mechanisms. They are known to be internalized and induce osteoclast apoptosis following resorption of bisphosphonate-coated bone (Flanagan and Chambers, 1989) while other studies have identified apoptosis of osteoclasts directly exposed to bisphosphonates. Vitté and colleagues (Vitté et al, 1996) and Sahni et al (1993) hypothesized that bisphosphonates affect osteoclast differentiation, survival and activity via osteoblast-produced intermediaries but did not identify these compounds. Yu et al subsequently demonstrated that UMR 106 cells express an osteoclast-inhibiting factor following bisphosphonate exposure and an osteoclast-stimulating factor after parathyroid hormone exposure (Yu et al, 1996). This supports the possibility that an imbalance in the relative expression of the osteoclast regulating genes by UMR 106 following bisphosphonate exposure might be responsible for the functional osteoclast impairment. This has important implications on our hypothesis that osteoclast-mediated tumour spread occurs in the local growth of osteosarcoma, and suggests the bisphosphonates may have an inhibitory effect on such growth in the in vivo setting.

DNA internucleosomal fragmentation, which is identified by a variety of commercial apoptosis assays, is an early event in apoptotic cell death (Arends et al, 1990). In this study we have demonstrated a significant increase in apoptosis in bisphosphonate-exposed cultures, but have not demonstrated a significant concentration-dependent effect with either bisphosphonate, following 4 hours of exposure. Although an increase is noted at $10^{-4}$ M pamidronate exposure, this concentration clearly caused marked cytotoxicity and had been previously excluded from proliferation and mRNA analyses. Despite no statistically significant dose-dependent changes in apoptosis in the first hours of bisphosphonate exposure our observation of increased apoptotic ratios on morphological assessment at 24 to 48 hours within the UMR 106-01 osteosarcoma cell cultures suggests ongoing effects.

During this early period of bisphosphonate exposure metabolic changes may be occurring within the cells which later direct the cell towards programmed cell death. Jilka et al demonstrated that a calvarial osteoblast culture and an osteosarcoma cell line culture exhibit background apoptosis, which may be modulated by various agents known to be involved in the regulation of Bone Modeling Units (BMUs) (Jilka et al, 1998). They were able to correlate this with TUNEL stain confirmation that a subpopulation of up to 50–70% of osteoblasts within BMUs may be lost to apoptosis. The mechanism by which bisphosphonates may induce osteoblast apoptosis and the relative potency or effect on osteosarcoma cells in vivo requires further investigation. The pathways by which the non-amino and aminobisphosphonates induce apoptosis in osteoclasts, breast cancer cells and macrophages are currently being studied and already the two broad groups of bisphosphonates have been found to differ in their mode of action (Luckman et al, 1998). The inhibitory effects of the amino-bisphosphonates on the mevalonate pathway in osteoclasts has now been well described (Russell et al, 1999; Rogers et al, 2000). However, the differing mode of action of clodronate (as described in osteoclasts) suggests that the effects we have noted on the osteosarcoma cells exposed to both clodronate and an amino-bisphosphonate are not attributable to an effect on the mevalonate pathway.

We have demonstrated a bisphosphonate-mediated down-regulation of OPN mRNA expression in an osteosarcoma cell line. OPN has been shown to mediate cell attachment and migration of malignant cells in culture (Oldberg et al, 1986; Majeska et al, 1993). It also has a well-defined role in protein mediated adherence between osteoblasts and the extracellular bone matrix (Butler, 1989; Helfrich et al, 1992; Majeska et al, 1993; Ross et al, 1993). The inhibition of OPN expression in osteoblasts by bisphosphonates has been reported by Sodek et al in mixed cultures of calvarial cell (Sodek et al, 1995). They demonstrated a reduction of OPN mRNA expression of approximately 50%, and to a much lesser degree reduced expression of type 1 collagen mRNA, but no alteration in the mRNA expression of bone sialoprotein and osteocalcin (Bone GLA protein). Osteoclasts and numerous non-bone tissues also express OPN (Denhardt and Guo, 1993; Merry et al, 1993; Dodds et al, 1995) and a role for OPN in cell to matrix binding has also been proposed in these cells (Reinholt et al, 1990). Recently, the importance of OPN for bone resorption in vivo has been demonstrated. Significantly less osteoclastic activity occurred on OPN-deficient bone discs implanted into the muscle of OPN knockout mice when compared to wild-type controls. Osteoclast activity was rescued by addition of recombinant OPN to the bone discs (Asou et al, 1999). These in vivo studies support

 © 2001 Cancer Research Campaign our studies and the proposal of Sodek et al that the OPN down-regulation in osteoblasts exposed to bisphosphonates may impair OPN-regulated osteoclastic bone resorption (Sodek et al, 1995).

Up-regulation of the OPN gene is found in primary metastatic tumours of various origins (Brown et al, 1994). Such up-regulation has been proposed to be a factor in tumorigenicity, possibly mediating tumour cell attachment to host tissues (Behrend et al, 1994, 1995; Gardner et al, 1994). It is known that transformed cell lines express higher levels of OPN than normal cells, although the significance of such expression remains unclear (Craig et al, 1988). Studies are required to demonstrate whether OPN inhibition observed in our study is mirrored in the primary tumours of in vivo models of osteosarcoma, and whether such inhibition is associated with an alteration in the biological activity of the tumour. The inhibition by nitrogen-containing bisphosphonates of breast cancer cell attachment to bone matrix in vitro (van der Pluijm et al, 1996; Boissier et al, 1997) bears out the role of these agents in reducing the bone metastatic burden of breast and prostate cancer now seen clinically. It has been suggested that the inhibiting effect of bisphosphonates on OPN expression in metastatic sub-clones of breast cancer may, in part, account for the reduction in metastatic disease (Orr et al, 1995).

We have demonstrated an inhibitory effect of bisphosphonates on cell proliferation as well as the expression of differentiated osteoblast function in a clonal rodent osteosarcoma cell line. Furthermore we have demonstrated an alteration in mRNA expression for OPN and RANKL, which may produce a net reduction in osteoclast function. If such osteoclast activity, or the phenotype of osteosarcomas, is altered by bisphosphonate exposure this may have specific implications in the advancement of osteosarcoma management. Future studies may be able to further elucidate the mechanisms by which the mRNA signal is regulated by the bisphosphonates and to ascertain whether post-transcriptional control of mRNA expression, or subsequent protein expression, is altered by bisphosphonates exposure. Additional studies are required to determine if such bisphosphonate effects are translated into the in vivo setting to functionally alter tumour-related osteoclast activity.

### ACKNOWLEDGEMENTS

Dr John Slavin, Department of Pathology, St Vincent's Hospital and Dr Elizabeth Williams, St Vincent's Institute for Medical Research for advice on apoptosis assessment. Professor T John Martin, St Vincent's Institute for Medical Research and Miss Monique Howard, Department of Orthopaedics, St Vincent's Hospital, for review of manuscript. Grants received from Royal Australasian College of Surgeons and the Anti-Cancer Council of Victoria.

### REFERENCES

Adami S (1997) Bisphosphonates in prostate carcinoma. *Cancer* **80**: 1674–1679

Arends MJ, Morris RG and Wyllie AH (1990) Apoptosis. The role of the endonuclease. *Am J Pathol* **136**: 593–608

Asou Y, Rittling SR, Yoshitake H, Denhardt DT and Noda M (1999) Osteopontin-deficient bone is defective in angiogenesis, osteoclast-recruitment and ectopic resorption. *Proceedings from the 21st Annual Meeting of the American Society for Bone and Mineral Research.*

Behrend EI, Craig AM, Wilson SM, Denhardt DT and Chambers AF (1994) Reduced malignancy of ras-transformed NIH 3T3 cells expressing antisense osteopontin RNA. *Cancer Res* **54**: 832–837

Behrend EI, Craig AM, Wilson SM, Denhardt DT and Chambers AF (1995) Expression of antisense osteopontin RNA in metastatic mouse fibroblasts is associated with reduced malignancy. *Ann N Y Acad Sci*, **760**: 299–301

Bloomfield DJ (1998) Should bisphosphonates be part of the standard therapy of patients with multiple myeloma or bone metastases from other cancers? An evidence-based review. *J Clin Oncol* **16**: 1218–1225

Boissier S, Magnetto S, Frappart L, Cuzin B, Ebetino FH, Delmas PD and Clezardin P (1997) Bisphosphonates inhibit prostate and breast carcinoma cell adhesion to unmineralized and mineralized bone extracellular matrices. *Cancer Res* **57**: 3890–3894

Brown LF, Papadopoulos Sergiou A, Berse B, Manseau EJ, Tognazzi K, Perruzzi CA, Dvorak, HF and Senger DR (1994) Osteopontin expression and distribution in human carcinomas. *Am J Pathol* **145**: 610–623

Butle, WT (1989). The nature and significance of osteopontin. *Connect Tissue Res* **23**: 123–136

Chan YL, Gutell R, Noller HF and Wool IG (1984) The nucleotide sequence of a rat 18 S ribosomal ribonucleic acid gene and a proposal for the secondary structure of 18 S ribosomal ribonucleic acid. *J Biol Chem* **259**: 224–230

Chikatsu N, Takeuchi Y, Tamura, Y, Fukumoto S, Yano K., Tsuda E, Ogata E and Fujita T (2000) Interactions between cancer and bone marrow cells induce osteoclast differentiation factor expression and osteoclast-like cell formation in vitro. *Biochem Biophys Res Commun* **267**: 632–637

Coleman RE and Purohit OP (1993) Osteoclast inhibition for the treatment of bone metastases. *Cancer Treat Rev* **19**: 79–103

Craig AM, Nemir M, Mukherjee BB, Chambers AF and Denhardt DT (1988) Identification of the major phosphoprotein secreted by many rodent cell lines as 2ar/osteopontin: enhanced expression in H-ras-transformed 3T3 cells. *Biochem Biophys Res Commun* **157**: 166–173

Denhardt DT and Guo X (1993) Osteopontin: a protein with diverse functions. *Faseb J* **7**: 1475–1482

Diel IJ, Solomayer EF, Costa SD, Gollan C, Goerner R, Wallwiener D, Kaufmann M and Bastert G (1998) Reduction in new metastases in breast cancer with adjuvant clodronate treatment. *N Engl J Med* **339**: 357–363

Dodds RA, Connor JR, James IE, Rykaczewski EL, Appelbaum E, Dul E and Gowen M (1995) Human osteoclasts, not osteoblasts, deposit osteopontin onto resorption surfaces: an in vitro and ex vivo study of remodeling bone. *J Bone Miner Res* **10**: 1666–1680

Evans CE and Braidman IP (1994) Effects of two novel bisphosphonates on bone cells in vitro. *Bone Miner* **26**: 95–107

Flanagan AM and Chambers TJ (1989) Dichloromethylenebisphosphonate (C12MBP) inhibits bone resorption through injury to osteoclasts that resorb C12MBP-coated bone. *Bone Miner* **6**: 33–43

Fleisch H (1993) New bisphosphonates in osteoporosis. *Osteoporos Int* 3 Suppl 2: S15–22

Galasko CS (1976) Mechanisms of bone destruction in the development of skeletal metastases. *Nature* **263**: 507–508

García Moreno C, Serrano S, Nacher M, Farré M, A, DI, Mariñoso, M, A, DI, Carbonell J, Mellibovsky L, Nogués X, Ballester J and Aubía, J (1998) Effect of alendronate on cultured normal human osteoblasts. *Bone* **22**: 233–239

Gardner HA, Berse B and Senger DR (1994) Specific reduction in osteopontin synthesis by antisense RNA inhibits the tumorigenicity of transformed Rat 1 fibroblasts. *Oncogene* **9**: 2321–2326

Goziotis A, Sukhu B, Torontali M, Dowhaniuk M and Tenenbaum HC (1995) Effects of bisphosphonates APD and HEBP on bone metabolism in vitro. *Bone* **16**: 317S–327S

Helfrich MH, Nesbitt SA, Dorey EL and Horton MA (1992) Rat osteoclasts adhere to a wide range of RGD (Arg-Gly-Asp) peptide-containing proteins, including the bone sialoproteins and fibronectin, via a beta 3 integrin. *J Bone Miner Res* **7**: 335–343

Horwood NJ, Elliott J, Martin TJ and Gillespie MT (1998) Osteotropic agents regulate the expression of osteoclast differentiation factor and osteoprotegerin in osteoblastic stromal cells. *Endocrinology* **139**: 4743–4746

Jilka RL, Weinstein RS, Bellido T, Parfitt AM and Manolagas SC (1998) Osteoblast programmed cell death (apoptosis): modulation by growth factors and cytokines. *J Bone Miner Res* **13**: 793–802

Kartsogiannis V, Zhou H, Horwood NJ, Thomas RJ, Hards DK, Quinn JMW, Niforas P, Ng KW, Martin TJ and Gillespie MT (1999) Localization of RANKL (Receptor Activator of NFKB Ligand) mRNA and Protein in Skeletal and Extraskeletal Tissues. *Bone* **25**: 525–534

Luckman SP, Hughes DE, Coxon FP, Graham R, Russell G and Rogers MJ (1998) Nitrogen-containing bisphosphonates inhibit the mevalonate pathway and prevent post-translational prenylation of GTP-binding proteins, including Ras. *J Bone Miner Res* **13**: 581–589

Majeska RJ, Port M and Einhorn TA (1993) Attachment to extracellular matrix molecules by cells differing in the expression of osteoblastic traits. *J Bone Miner Res* **8**: 277–289

© 2001 Cancer Research Campaign 

Martin TJ, Ingleton PM, Underwood JC, Michelangeli VP, Hunt NH, and Melick RA (1976) Parathyroid hormone-responsive adenylate cyclase in induced transplantable osteogenic rat sarcoma. *Nature*, **260,** 436–438

Merry K, Dodds R, Littlewood A and Gowen M (1993) Expression of osteopontin mRNA by osteoclasts and osteoblasts in modelling adult human bone. *J Cell Sci* **104**: 1013–1020

Oldberg A, Franzén A and Heinegård D (1986) Cloning and sequence analysis of rat bone sialoprotein (osteopontin) cDNA reveals an Arg-Gly-Asp cell-binding sequence. *Proc Natl Acad Sci USA* **83**: 8819–8823

Orr FW, Sanchez-Sweatman OH, Kostenuik P and Singh G (1995) Tumor-bone interactions in skeletal metastasis. *Clin Orthop* 19–33

Plotkin LI, Weinstein RS, Parfitt AM, Roberson PK, Manolagas SC and Bellido T (1999) Prevention of osteocyte and osteoblast apoptosis by bisphosphonates and calcitonin. *J Clin Invest* **104**: 1363–1374

Reinholt FP, Hultenby K, Oldberg A and Heinegard D (1990) Osteopontin – a possible anchor of osteoclasts to bone. *Proc Natl Acad Sci USA* **87**: 4473–4475

Rogers MJ, Gordon S, Benford HL, Coxon FP, Luckman SP, Monkkonen J and Frith JC (2000) Cellular and molecular mechanisms of action of bisphosphonates. *Cancer* **88**: 2989–2994

Ross FP, Chappel J, Alvarez JI, Sander D, Butler WT, Farach-Carson MC, Mintz KA, Robey PG, Teitelbaum SL and Cheresh DA (1993) Interactions between the bone matrix proteins osteopontin and bone sialoprotein and the osteoclast integrin alpha v beta 3 potentiate bone resorption. *J Biol Chem* **268**: 9901–9907

Russell RG, Rogers MJ, Frith JC, Luckman SP, Coxon FP, Benford HL, Croucher PI, Shipman C and Fleisch HA (1999) The pharmacology of bisphosphonates and new insights into their mechanisms of action *J Bone Miner Res* **14**: 53–65

Sahni M, Guenther HL, Fleisch H, Collin P and Martin TJ (1993) Bisphosphonates act on rat bone resorption through the mediation of osteoblasts. *J Clin Invest* **91**: 2004–2011

Sato M, Grasser W, Endo N, Akins R, Simmons H, Thompson DD, Golub E and Rodan GA (1991) Bisphosphonate action. Alendronate localization in rat bone and effects on osteoclast ultrastructure. *J Clin Invest* **88**, 2095–2105

Senaratne SG, Pirianov G, Mansi JL, Arnett TR and Colston KW (2000) Bisphosphonates induce apoptosis in human breast cancer cell lines. *Br J Cancer* **82**: 1459–1468

Shipman CM, Rogers MJ, Apperley JF, Russell RG and Croucher PI (1997) Bisphosphonates induce apoptosis in human myeloma cell lines: a novel anti-tumour activity. *Br J Haematol* **98**: 665–672

Simonet WS, Lacey DL, Dunstan CR, Kelley M, Chang MS, Luthy R, Nguyen HQ, Wooden S, Bennett L, Boone T, Shimamoto G, DeRose M, Elliott R, Colombero A, Tan HL, Trail G, Sullivan J, Davy E, Bucay N, Renshaw-Gegg L, Hughes TM, Hill D, Pattison W, Campbell P, Boyle WJ, et al (1997) Osteoprotegerin: a novel secreted protein involved in the regulation of bone density. *Cell* **89**: 309–319

Sodek J, Chen J, Nagata T, Kasugai S, Todescan R, Jr, Li IW and Kim RH (1995) Regulation of osteopontin expression in osteoblasts. *Ann N Y Acad Sci*, **760**: 223–241

Tan PL, Katz JM, Ames R, Caughey DE, Gray HD, Ibbertson HK and Watson JD (1988) Aminobisphosphonate inhibition of interleukin-1-induced bone resorption in mouse calvariae. *Arthritis Rheum* **31**: 762–768

Thiébaud D, Leyvraz S, von Fliedner V, Perey L, Cornu P, Thiébaud S and Burckhardt P (1991) Treatment of bone metastases from breast cancer and myeloma with pamidronate. *Eur J Cancer* **27**: 37–41

van der Pluijm G, Vloedgraven H, van Beek E, van der Wee- Pals L, C, Lö and Papapoulos S (1996). Bisphosphonates inhibit the adhesion of breast cancer cells to bone matrices in vitro. *J Clin Invest* **98**: 698–705

Vitté C, Fleisch H and Guenther HL (1996). Bisphosphonates induce osteoblasts to secrete an inhibitor of osteoclast-mediated resorption. *Endocrinology* **137**: 2324–2333

Wong BR, Rho J, Arron J, Robinson E, Orlinick J, Chao M, Kalachikov S, Cayani E, Bartlett FS, 3rd, Frankel WN, Lee SY and Choi Y (1997). TRANCE is a novel ligand of the tumor necrosis factor receptor family that activates c-Jun N-terminal kinase in T cells *J Biol Chem* **272**: 25190–25194

Yasuda H, Shima N, Nakagawa N, Mochizuki SI, Yano K, Fujise N, Sato Y, Goto M, Yamaguchi K, Kuriyama M, Kanno T, Murakami A, Tsuda E, Morinaga T and Higashio K (1998a). Identity of osteoclastogenesis inhibitory factor (OCIF) and osteoprotegerin (OPG): a mechanism by which OPG/OCIF inhibits osteoclastogenesis in vitro. *Endocrinology*, **139**: 1329–1337

Yasuda H, Shima N, Nakagawa N, Yamaguchi K, Kinosaki M, Mochizuki S, Tomoyasu A, Yano K, Goto M, Murakami A, Tsuda E, Morinaga T, Higashio K, Udagawa N, Takahashi N and Suda T (1998b). Osteoclast differentiation factor is a ligand for osteoprotegerin/osteoclastogenesis-inhibitory factor and is identical to TRANCE/RANKL. *Proc Natl Acad Sci USA* **95**: 3597–3602

Yoshida H, Hayashi S, Kunisada T, Ogawa M, Nishikawa S, Okamura H, Sudo T, Shultz LD and Nishikawa S (1990). The murine mutation osteopetrosis is in the coding region of the macrophage colony stimulating factor gene. *Nature* **345**: 442–444

Yu X, Scholler J and Foged NT (1996) Interaction between effects of parathyroid hormone and bisphosphonate on regulation of osteoclast activity by the osteoblast-like cell line UMR-106. *Bone* **19**: 339–345

*© 2001 Cancer Research Campaign*
